# Effect of angiotensin II receptor blockers on efficacy and safety of Camrelizumab plus chemotherapy in the first-line therapy for advanced non-small cell lung cancer (ARMOR I): a protocol for a prospective, real-world, multicenter, intervention clinical trial

**DOI:** 10.3389/fimmu.2025.1668782

**Published:** 2025-11-11

**Authors:** Zeyu Wang, Huiyu Wang, Rui Hou, Huning Jiang, Hanfang Fan, Yuhan Zhang, Yichao Zhu, Yun Cai, Jie Mei, Junying Xu

**Affiliations:** 1 Departments of Oncology, The Affiliated Wuxi People’s Hospital of Nanjing Medical University, Wuxi Medical Center, Nanjing Medical University, Wuxi, China; 2 Department of Physiology, School of Basic Medical Sciences, Nanjing Medical University, Nanjing, China; 3 Central Laboratory Changzhou Jintan First People’s Hospital The Affiliated Jintan Hospital of Jiangsu University, Changzhou, Jiangsu, China; 4 The First Clinical Medicine College, Nanjing Medical University, Nanjing, China

**Keywords:** arb, Camrelizumab, immunotherapy, collagen, clinical trial, protocol

## Abstract

**Background:**

Lung cancer, particularly non-small cell lung cancer (NSCLC), remains a leading cause of cancer-related mortality worldwide. While immune checkpoint blockade (ICB), such as PD-1/PD-L1 inhibitors, have revolutionized treatment for advanced NSCLC, not all tumor patients respond to ICB therapy. Our recent investigations highlight the role of collagens synthesized by cancer-associated fibroblasts (CAFs) in immune evasion. Angiotensin II receptor blocker (ARB) has shown potential in reshaping the tumor microenvironment (TME) by inhibiting collagens, making tumors more susceptible to immunotherapy. This study aims to evaluate the effect of ARB on the efficacy and safety of Camrelizumab, an anti-PD-1 antibody, in combination with chemotherapy for first-line treatment of advanced NSCLC.

**Methods:**

The ARMOR I trial is a prospective, real-world, multicenter, intervention clinical study designed to assess the synergistic effect of ARBs on Camrelizumab plus chemotherapy in patients with advanced NSCLC. Eligible patients include those with stage IV or unresectable locally advanced NSCLC, who have been diagnosed with hypertension and are receiving standard treatment for it. The study will enroll approximately 180 patients over a 12-month recruitment period, with a 12-month follow-up phase. The primary endpoint is the objective response rate (ORR), with secondary endpoints including progression-free survival (PFS), overall survival (OS), and safety.

**Discussion:**

The interplay between collagens, ARB, and cancer is still complex and worth further study. ARMOR I will provide crucial preliminary data on ARB’s role in first-line therapy for advanced NSCLC. The potential application of ARB in other tumor types may also become an important area to explore.

## Background

Lung cancer ranks as the second most diagnosed cancer and is the primary contributor to cancer-related mortality globally (1). Approximately 85% of all lung cancer cases are classified as non-small cell lung cancer (NSCLC), representing a significant health challenge in oncology (2, 3). The epidemiology of NSCLC indicates a high incidence and mortality rate, with factors such as tobacco use, family history, and chronic lung diseases being critical risk factors (4). Immune checkpoint inhibitor (ICB), such as PD-L1 antibody, stimulates the immune system to kill tumor cells by blocking PD-1/PD-L1 immune checkpoint pathway (5). Based on the extended follow-up data from the phase III CameL and CameL-sq trials, Camrelizumab, a humanized anti-PD-1 monoclonal antibody, combined with carboplatin and pemetrexed, showed sustained and clinically meaningful improvements in overall survival (OS) and progression-free survival (PFS) compared with chemotherapy alone in patients with previously untreated advanced non-squamous NSCLC without EGFR or ALK alterations (6, 7).

However, ICB is not a cure-all for cancer because not all tumor patients respond to ICB therapy (8–10). Previous study has established a pan-cancer immuno-collagenic subtyping strategy to stratify patients who respond favorably to ICB therapy according to collagen deposition and tumor immune microenvironment (TIME), and identifies a refractory cancer subtype with high collagen activity and low immune infiltration, which is defined as armored and cold subtype with the lowest immunotherapeutic response rate (11). CAFs are capable of secreting many extracellular matrix components (ECM), and physical exclusion consisting of collagens downregulate T cell effector function and weaken tumor immunity (12–14). Collagens and related factors have been demonstrated to be potential markers for immunotherapy resistance (15, 16).

Tumor patients often receive combination therapy to manage pre-existing comorbid conditions or to alleviate side effects from antitumor therapy (17). Polypharmacy is prevalent in tumor patients, with a study indicating it affects up to 84% of them (18). Angiotensin receptor blocker (ARB) is the most frequently prescribed class of antihypertensive drugs accounting for 51.6% of all antihypertensive drugs (19). Accumulating data have indicated that the renin‐angiotensin system (RAS) facilitates malignancy and correlates with poor patient outcomes for various cancers (14). Angiotensin II (Ang II), a key effector of the RAS, has been implicated in promoting tumor growth and immune evasion, highlighting its potential as a therapeutic target (20, 21). ARB is reported to have an anti-pulmonary fibrotic function (22). In our preclinical study, angiotensin II receptor 1 (AGTR1) is overexpressed in armored and cold tumors and primarily expressed in CAFs, predicting poor immunotherapeutic response. Mechanistically, AGTR1 inhibitor ARB inhibits collagen expression in cancer-associated fibroblasts (CAFs) via suppressing the RhoA-YAP axis. Consequently, ARB shapes a soft and hot TME and improves therapeutic efficacy of ICB (23). A retrospective study involving 597 patients with advanced solid tumors demonstrated that the concomitant use of ARB was associated with a significant improvement in the objective response rate (ORR) among patients receiving ICB (24). Similar findings were also observed in a retrospective study presented at ESMO 2021 that contained 127 patients with NSCLC receiving ICB therapy, and demonstrated that the concomitant use of ARB was associated with an increase in the ORR.

The combination medication of ARB and ICB emerges as a promising treatment option that can prolong the survival of tumor patients with poor response to ICB. The benefits of ARB derived from the ability of inhibiting collagen and promoting immune cell infiltration warrant further examination in a prospective, controlled study. To date, no studies have evaluated the effect of ARB in the first-line ICB therapy for advanced NSCLC. ARMOR I is a prospective, real-world, multicenter, intervention clinical study intended to observe the effect of ARB on the efficacy and safety of Camrelizumab plus chemotherapy in the first-line therapy for advanced NSCLC. The ARMOR I trial will investigate whether ARB sensitizes Camrelizumab plus chemotherapy in the first-line treatment of advanced NSCLC.

## Methods

### Study design

**Figure 1:**
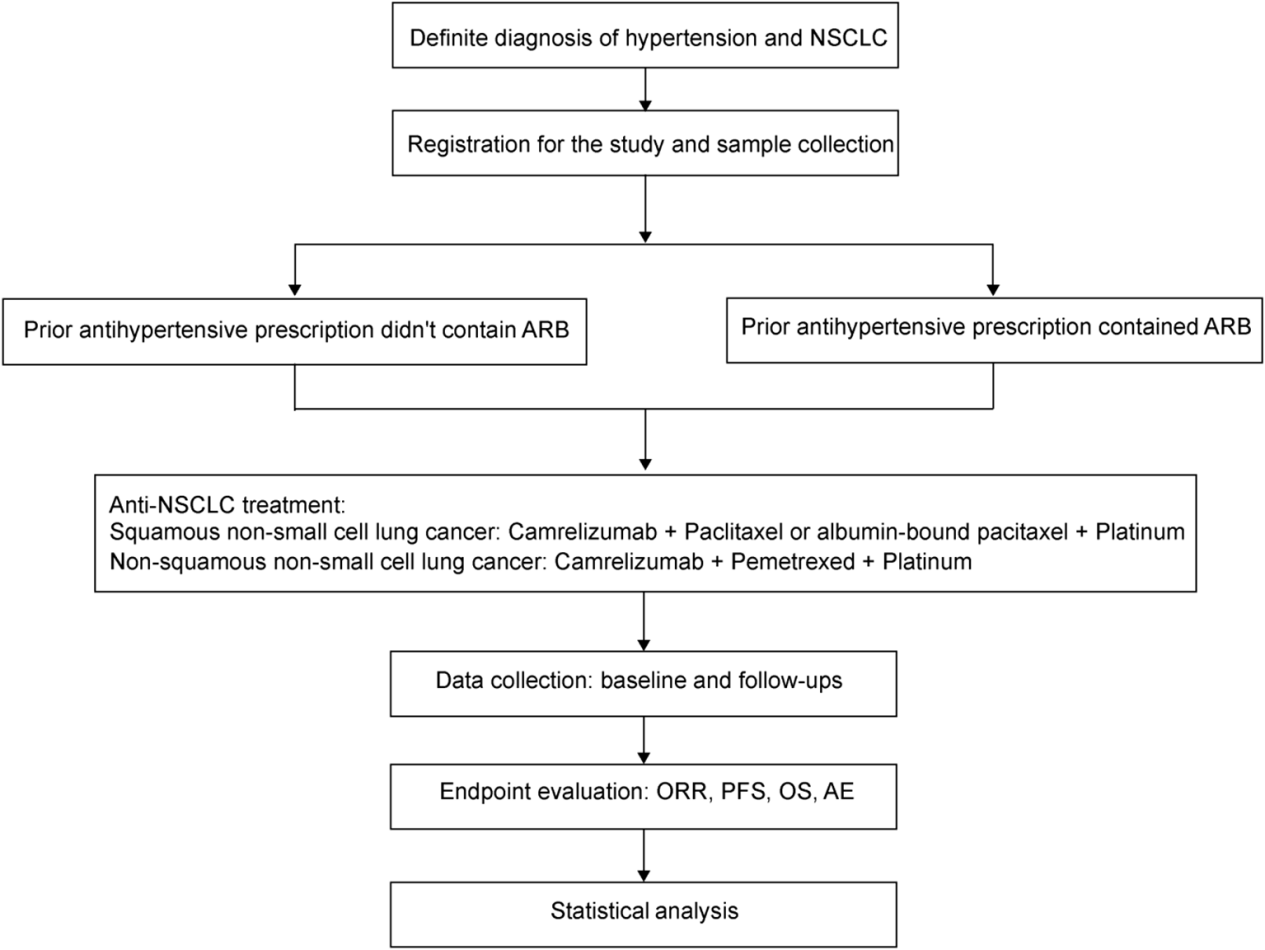
Study plan of the ARMOR I clinical trial.

ARMOR I is a prospective, real-world, multicenter, intervention clinical study intended to observe the effect of ARB on the efficacy and safety of Camrelizumab plus chemotherapy in the first-line therapy for advanced NSCLC (**Figure 1**). The study aims to preliminarily evaluate the sensitization effect of ARB on Camrelizumab plus chemotherapy, laying the foundation for conducting further randomized controlled trials (RCTs). Enrollment duration is 24 months. The specific study plan is presented in **Figure 1**.

The online tool Epitools (http://epitools.ausvet.com.au) was used to calculate the sample size with a two-sided test significance level of 0.05 and a statistical power of 80%. This calculation was based on the ORR observed in our previous retrospective study: 47.222% for hypertensive NSCLC patients receiving concomitant ARB medication, and 24.691% for those not receiving concomitant ARB medication (23). Thus, the total sample size was calculated as 158 subjects under a 1:1 enrollment ratio. In addition, to account for a potential 10% dropout rate during the follow-up period, the total planned sample size is 174 subjects.

### Patient selection

#### Inclusion criteria

Patients meeting all the following inclusion criteria are eligible for enrollment into the study:

1. Voluntarily agree to join the project and sign an informed consent form.
2. Prior diagnosis of hypertension which meet the standard of drug intervention in 2024 Chinese guidelines for the management of hypertension.
3. Histopathologically or cytologically confirmed diagnosis of NSCLC.
4. Men and women aged 18 to 75 years.
5. Clinical stage determined by the Eighth Edition AJCC Cancer Staging System as stage IV or unresectable locally advanced stage (IIIb-IIIc).
6. No prior systemic anti-tumor therapy for advanced NSCLC. Subjects with recurrent NSCLC who have received adjuvant or neoadjuvant therapy (chemotherapy, radiation, or other treatment) can be enrolled if the interval between last treatment and recurrence is more than 6 months.
7. At least 1 measurable lesion according to Response Evaluation Criteria in Solid Tumors (RECIST v1.1).
8. An expected survival of at least 12 weeks.
9. Agree to provide a tumor tissue sample when or after the diagnosis of advanced NSCLC. The sample should be an archived or freshly obtained 4-6 µm unstained sections
10. ECOG performance status score of 0-1.
11. The function of vital organs should meet the following requirements (excluding the use of any blood components and cell growth factors during screening):
  - Absolute neutrophil count (ANC) ≥ 1.5×10^9^/L
  - Platelet count (PLT) ≥ 100×10^9^/L
  - Hemoglobin (HB) ≥ 9g/dL
  - Serum albumin ≥ 3g/dL
  - Thyroid-stimulating hormone (TSH) ≤ upper limit of normal (ULN) (subjects with abnormal TSH levels should be evaluated by T3 and T4 levels. If T3 and T4 levels are normal, subjects can be enrolled)
  - Bilirubin ≤ 1.5×ULN
  - Alanine aminotransferase (ALT) and aspartate aminotransferase (AST) ≤ 2.5×ULN
  - Alkaline phosphatase (AKP) ≤ 2.5×ULN
  - Creatinine (Cr) ≤ 1.5×ULN
  - Creatinine clearance rate ≥ 60mL/min.
12. Female subjects with fertility and male subjects whose partners are women of childbearing age must use effective contraceptive measures during the study treatment and for at least 6 months after the last treatment.

#### Exclusion criteria

Patients meeting any of the following criteria are excluded from the study:

1. No prior anti-PD-1/PD-L1 or other forms of immunotherapy.
2. Known active central nervous system metastasis without surgery or radiotherapy.
3. History of other malignant tumors in the past 5 years, excluding cured basal cell carcinoma of the skin and various cancers *in situ*.
4. Receipt of any anti-tumor treatment within 4 weeks prior to joining the study.
5. Poor compliance and inability to adhere to medication as per the doctor’s advice.
6. Pregnant or breastfeeding female (females of childbearing age must have a negative pregnancy test within 14 days before the first dose, if positive, the pregnancy must be excluded by ultrasound)
7. Suffering from gastrointestinal dysfunction or gastrointestinal disease that can significantly affect the absorption of the study drug (e.g., uncontrolled ulcerative disease, nausea, vomiting, diarrhea, malabsorption syndrome, or small bowel resection).
8. Patients with clinical symptoms such as ascites, pleural effusion, and pericardial effusion in need of drainage during the baseline period, or those who have undergone drainage of serous cavity within 4 weeks before the first dose.
9. History of immunodeficiency, known HIV, other acquired or congenital immunodeficiency diseases, or history of organ transplantation.
10. Patients who have undergone major surgeries or have significant trauma within 4 weeks before starting treatment, or who will require major surgical treatment.
11. Any illness that could jeopardize the safety of patients and their compliance in the study according to the judgement of the researcher, such as accelerated hypertension, hypertensive encephalopathy, diabetes, thyroid disease, or co-infection with active hepatitis B and other active infections, etc.
12. Unable to understand or follow the study instructions and requirements
13. Unsuitable to participate in this study as judged by the researchers.

#### Interventions

All patients are subjected to the following treatment regimens:

**Squamous non-small cell lung cancer:** Camrelizumab **+** Paclitaxel or albumin-bound paclitaxel + Platinum

**Non-squamous non-small cell lung cancer:** Camrelizumab + Pemetrexed + Platinum

Patients are divided into the following cohorts according to prior received antihypertensive drugs:

**Cohort 1:** Patients in this cohort are treated with antihypertensive drugs that do not contain ARBs, or their condition met the criteria for drug intervention as outlined in the 2024 Chinese guidelines for the management of hypertension, but no drug intervention was implemented. The treatment plan shouldn’t be changed due to this study. If ARBs have been used in the past, the last dose must have been administered more than 3 months prior to the start of immunotherapy.

**Cohort 2**: Patients in this cohort are treated with antihypertensive drugs that contain ARBs. Specifically, ARBs have been used based on the recommendations of cardiologists and the preferences of the patients. The duration of ARB medication should exceed 3 months prior to the initiation of immunotherapy. The treatment plan shouldn’t be changed due to this study.

To reduce potential heterogeneity from different ARBs, each drug could be analyzed separately, and a subgroup analysis could be conducted to compare the outcomes of different ARBs.

### Combined medication

Any combination therapy, operation, or other drugs that the researcher deems necessary for the clinical needs of the subject must be recorded. This includes vaccines (both over-the-counter and prescription drugs), vitamins, and Chinese herbal supplements, as well as other specific types of treatments that subjects have received since the initiation of screening period or during the study period, which includes a follow-up period of 40 days (+7 days) after the last dose of the study intervention drugs. It is essential to document the reason for combined medication, dosing dates (including start and end dates), and dosing information, which encompasses dosage and frequency of administration. It should be emphasized that this study is observational in nature, and any treatment plan should not be altered due to participation in this study. All combined medications are necessary for clinical treatment.

### Data collection

All data will be collected using an electronic data capture system through direct data entry by site clinical and research staff. Follow-up data will be collected over the course of any standard of care visits occurring after completion of study treatment.

1. **History taking:** Demographic information should be collected and recorded as per protocol within 14 days before the start of treatment, including age, sex, nation, smoking history and complete medical history containing tumor history, past medical history, menstrual history, family history, etc.
2. **Pathological diagnosis:** Detailed pathological diagnosis information and light microscopic images of HE-stained tumor tissues should be recorded and collected.
3. **Physical examination:** A physical examination shall be carried out according to the following schedule and recorded, including vital signs (blood pressure, heart rate, temperature and respiratory rate), height, weight, Eastern Cooperative Oncology Group performance status (ECOG PS), location and number of tumor metastasis and inspection of each system.
4. **ECG:** An ECG examination should be performed according to the needs of clinical diagnosis and treatment.
5. **Imaging examination:** Imaging examinations should be performed according to the needs of clinical diagnosis and treatment, including:
  - Chest CT: A chest CT (plain or enhanced) needs to be performed at baseline, and patients should be reviewed according to the prescribed schedule when evaluating curative effect.
  - Abdominal CT: An abdominal CT needs to be performed at baseline, and patients should be reviewed according to the prescribed schedule when evaluating curative effect.
  - Osseous metastasis related examination: If no corresponding symptoms appear, the relevant examination can be omitted at baseline. If symptoms associated with osseous metastasis are present, a total body emission computed tomography (ECT) scan of bones can be used to detect osseous metastases. If further diagnosis is required, an MRI examination of the corresponding location of bone metastasis can be performed, and patients should be reviewed according to the prescribed schedule when evaluating curative effect.
  - Cranial imaging examination: If no corresponding symptoms appear, the relevant examination can be omitted at baseline. If symptoms associated with brain metastasis are present, a cranial MRI scan and total body scan of the bones can be used to detect brain metastases. An MRI can be performed at any time after the initiation of the trial medication if intracranial lesions are suspected, and patients should be reviewed according to the prescribed schedule.
  - Other imaging examinations: Other imaging examination, such as PET-CT, can be performed according to the needs of clinical diagnosis and treatment.
6. **Laboratory test:** Peripheral blood cell count, blood biochemistry, routine urinalysis and other tests can be performed according to the needs of clinical diagnosis and treatment.
7. **Research schedule:** Enrollment began in Feb. 2025 and is currently in progress with the aim of completing enrollment by Feb. 2027.

### Study endpoints

**Primary endpoints:** The objective response rates (ORR) are defined as the sum of the partial response (PR) and complete response (CR) rates, as per the Response Evaluation Criteria In Solid Tumors (RECIST) 1.1.

The progression of target lesions (TL) should be evaluated against those with the minimum tumor load (such as the minimum sum of length and diameter after the start of the study). In addition to disease progression, other tumor remission indexes (CR, PR and SD) are compared with the baseline level. If the researcher or client is uncertain whether the disease is progressing, especially in cases where the non-target lesions are relieved and new lesions appear, the treatment may continue until the next evaluation, or progression can be re-evaluated as soon as clinically necessary.

The evaluation methods for tumor baseline include CT or MRI of chest and abdomen, and follow-up evaluations of the same lesion must utilize the same examination method. Other suspicious lesions can also be examined and evaluated during the baseline and follow-up examinations. Following the baseline evaluation, all patients who meet the criteria should be re-evaluated at the end of every two cycles (refer to the evaluation schedule for details) until disease progression as defined by RECIST v1.1.

If patients withdraw from treatment (and/or receive other treatment) before disease progression occurs, they should still be followed up every 6 weeks, until disease progression as defined by RECIST v1.1. If the progression is re-evaluated and confirmed, the date of disease progression will be considered the initial examination date.

If disease progression is determined based on non-target lesions, it can only be classified as progression when the non-target lesions exhibit obvious deterioration and the overall tumor burden level (including both target and non-target lesions) has significantly increased to the extent that treatment needs to be terminated, even if the target lesion is classified as SD or PR. A slight enlargement of one or more non-target lesions does not meet the criteria for obvious progression.

**Secondary endpoints**

1. **Overall survival (OS):** OS is defined as the as the length of time from the date when the patients receive trial medication to the date of death.
2. **Progression-free survival (PFS):** PFS is defined as the time from receiving trial medication until the first occurrence of disease progression or death, or the date of the last tumor assessment if no disease progression or death occurs. Before patients receiving trial medication experience a PFS event, efficacy will be evaluated every 2 cycles of treatment. After experiencing PFS events, patients will be followed up for survival information every 3 months. The management of patients (the initial PFS analysis) should be determined by researchers’ assessment according to RECIST v1.1, but it is necessary to continue collecting all imaging examination results, including those obtained outside the stipulated time.
3. **Adverse events** (AE): The incidence and severity of adverse events and severe adverse events (SAE) are evaluated according to the Common Terminology Criteria for Adverse Events (CTCAE 5.0). Given the potential effect of ARB on vascular endothelial cells, detailed records of reactive cutaneous capillary endothelial proliferation (RCCEP) are needed to evaluate whether ARB can help alleviate RCCEP. RCCEP will be assessed every cycle using clinical examination. Grading and management will follow CTCAE v5.0. Suspected RCCEP will be referred to dermatology for further evaluation and treatment if necessary.

### Biological sample retention

**Figure 2:**
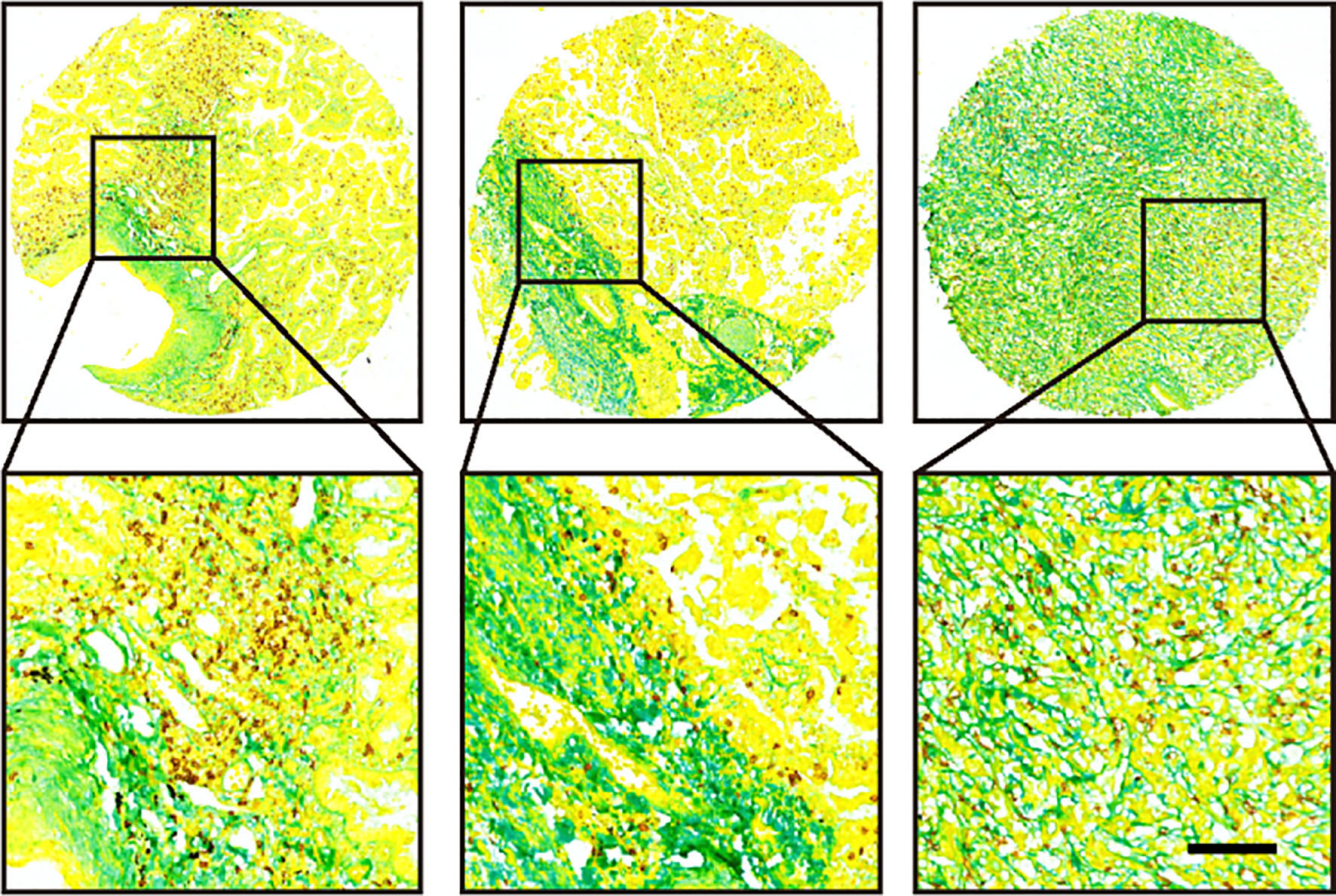
Representative images of three tumor samples after multiplexed staining by the new method.

First, one piece of 4 µm paraffin-embedded unstained section collected pre-immunotherapy, which will be submitted for multiplexed staining of collagen and CD8. A novel multiplexed staining protocol was developed for detecting the levels of collagen and CD8^+^ T cell in paraffin-embedded tissues. Based on this method, we can achieve simultaneous detection of collagen and T cells on the same tissue section (**Figure 2**). Briefly, sections underwent deparaffinization in xylene (3 × 10 min) and rehydration through graded ethanol (100%, 95%, 85%, 70%; 5 min each) followed by PBS washes (3 × 3 min). For epitope retrieval, sections were placed in preheated boiling EDTA buffer (10 min, 95–100 °C), allowed to cool naturally, and rinsed with PBS (3 × 3 min). To block endogenous peroxidase activity, sections were treated with a solution of 3% H_2_O_2_-methanol (10 min, RT), and nonspecific sites were blocked with 10% goat serum (20 min, RT) in a humid chamber. After incubation with anti-CD8 primary antibody (GT2112, Suzhou Abcarta; 4 °C overnight) and secondary antibody (30 min, RT), DAB development (5–10 min, RT) was microscopically monitored and terminated with distilled water. The sections were counterstained with hematoxylin (5 min), blued in 1% ammonia solution, then subjected to aniline blue staining (G1476, Beijing Solarbio; 5 min, RT) followed by strictly timed differentiation in 0.5% glacial acetic acid (within 30 sec). Dehydration involved 95% ethanol (5 sec), 100% ethanol (5 sec + 30 sec), xylene clearance (2 × 1 min), and finally mounted with neutral resin.

In addition, a total of 5 mL of full blood must be collected prior to the administration of immunotherapy and within three days before the first efficacy evaluation. The detection indicators include TGFβ, COL1, IFN-γ, and other markers related to fibrosis and immune status. The full blood should be centrifuged to extract serum and stored at -80°C.

### Statistical analysis

A Chi-square test will be used to compare the ORR between Cohort 1 and 2. Continuous biomarker scores will be dichotomized at the median or using optimal cut-off values determined by maximally selected rank statistics for survival outcomes. Kaplan-Meier method will be used to plot survival curves of PFS and OS for each cohort. Comparisons of survival distributions between Cohort 1 and 2 will be made using a log-rank test. Adverse events will be summarized by frequency and severity, classified according to CTCAE 5.0. The incidence of adverse events between groups will be compared using Chi-square tests or Fisher’s exact tests as appropriate. To control for potential confounding factors, we will collect comprehensive baseline characteristics. Propensity score matching (PSM) and multivariate regression analyses will be employed to adjust for imbalances and better estimate the effect of ARB as well. In addition, the expression of collagen and CD8 in tumor tissues and serum expression of TGFβ, COL1, IFN-γ, and other markers related to fibrosis and immune status at baseline will be compared using Student’s t-test or Mann-Whitney test. In addition, the correlation between these biomarkers will be assessed using Spearman’s correlation analysis.

## Discussion

ICBs have revolutionized the treatment landscape for advanced NSCLC, providing patients with improved survival compared to traditional chemotherapy. Despite this progress, the effectiveness of ICB therapy varies significantly among patients, with some patients exhibiting resistance linked to the characteristics of TME (8, 9). ARMOR I aims to address this gap by evaluating the efficacy and safety of ARB combined with Camrelizumab and chemotherapy in first-line treatment for advanced NSCLC, exploring the potential benefits of this novel combination therapy.

Collagens serve not only as structural elements within TME, but also as active agents in modulating immune responses. The interplay between collagens, ARB, and cancer is complex, with emerging evidence supporting the potential of ARB to alter TME positively. Recent studies have elucidated various mechanisms through which collagens mediate immunosuppressive effects. The latest studies have demonstrated that tumor-associated macrophages (TAMs) exhibit altered functions in response to collagen density within the TME. High collagen density has been shown to enhance the immunosuppressive phenotype of TAMs, thereby reducing the efficacy of cytotoxic T cell responses and contributing to poor patient prognosis in several cancers, including breast cancer and pancreatic cancer (25, 26). In addition, collagens can promote immunotherapy resistance in lung cancer through LAIR1-dependent CD8+ T cell exhaustion. Collagens activate leukocyte-specific collagen receptor LAIR1 on CD8+ T cells, then LAIR1 induces T cell exhaustion through SHP-1 (15). Furthermore, CAFs, the principal producers of collagens within solid tumors, are also able to promote cancer cell progression and immune evasion through secretion of immunomodulatory molecules, physical interaction with immune cells and remodeling of the extracellular matrix (27). ARMOR I is designed to validate the above hypothesis and provide real-world data to support it.

ARB, a well-known antihypertensive drug, has been found to reduce CAFs activation and collagen deposition via TGF-β1-dependent pathways in tumors (28). One significant study demonstrated that the ARB telmisartan can inhibit the development of transient hypoxia in tumors, which is crucial for enhancing the efficacy of radiation therapy. This research found that telmisartan reduces collagen deposition produced by CAFs, thereby improving tumor blood flow and decreasing hypoxic regions within tumors. Consequently, tumors treated with telmisartan exhibited a more favorable response to radiation therapy, suggesting that ARB may modify the tumor microenvironment to enhance therapeutic outcomes (29). Similarly, our previous finding also indicated that ARB can inhibit collagen expression in CAFs and reverse the phenotypic characteristics of tumors, making them more amenable to ICB (23).

Beyond modulating collagen deposition, ARBs are known to exert significant effects on tumor vasculature. Existing studies have provided evidence that ARBs can modulate tumor vasculature. For instance, research has shown that candesartan induces vascular normalization in prostate cancer xenografts by downregulating VEGF expression and reducing vascular permeability. This process enhances endothelial barrier function, which can positively influence the tumor microenvironment. These changes may indirectly affect the efficacy of subsequent treatments, particularly by improving drug delivery in chemotherapy and enhancing responses to immunotherapy (30). Thus, the potential benefit of ARBs observed in our study may be attributed not only to collagen remodeling but also to improved vascular function, ultimately enhancing the efficacy of Camrelizumab and chemotherapy.

RCCEP is the most common adverse event related to Camrelizumab (31). RCCEP is characterized by the abnormal growth of capillary endothelial cells in the skin. The pathogenesis of RCCEP involves a complex interplay of factors, including endothelial cell activation, proliferation, and angiogenesis, which can be driven by cytokines and growth factors such as VEGF and FGF. One study demonstrated that ARB, such as telmisartan and azilsartan, significantly increased endothelial NO production and improved vascular function independent of their blood pressure-lowering effects. This suggests a pleiotropic effect of ARB, where they promote endothelial health by enhancing NO bioavailability and mitigating the adverse effects of angiotensin II on endothelial cells (32, 33). Mechanistically, ARB has the potential to prevent RCCEP.

ARMOR I is the first prospective, real-world, multicenter, intervention clinical trial designed to systematically evaluate the role of ARB in enhancing the efficacy and safety of Camrelizumab plus chemotherapy in the first-line therapy for advanced NSCLC. By comparing ARB-treated and non-ARB-treated cohorts, we aim to explore whether ARBs can act as a synergistic therapy to improve the ORR and clinical outcomes of Camrelizumab plus chemotherapy. Furthermore, the study will be the first to assess the potential alleviating effects of ARB on treatment-related adverse events, such as RCCEP.

In future research, the main challenge will be how to define an appropriate control group, since patients who receive ARB therapy often differ in baseline characteristics and treatment context from those who do not. PSM offers a useful approach to balance these differences and reduce potential confounding. Another promising strategy is the use of real-world data to construct matched cohorts with comparable clinical features, which can provide a more reliable framework for validating the observed associations.

The results of ARMOR I will provide critical preliminary evidence for the clinical application of ARBs in combination with Camrelizumab and chemotherapy, setting the stage for future researches. If the combination therapy demonstrates significant efficacy and safety advantages in this study, ARB could become a key element in the first-line treatment for advanced NSCLC. Additionally, future research could further optimize patient stratification strategies, such as identifying patients most likely to benefit from the combination therapy based on TME characteristics or biomarker levels. Moreover, as research progresses, the potential application of ARB in other tumor types may also become an important area to explore.

## Conclusions

In summary, ARMOR I will not only provide critical clinical evidence for the role of ARBs in cancer immunotherapy, but also hold the potential to bring new hope to the personalized treatment of NSCLC.
